# Use of Local Antibiogram Data and Antimicrobial Importance Ratings to Select Optimal Empirical Therapies for Urinary Tract Infections in Dogs and Cats

**DOI:** 10.3390/antibiotics9120924

**Published:** 2020-12-18

**Authors:** Ri Scarborough, Kirsten Bailey, Bradley Galgut, Adam Williamson, Laura Hardefeldt, James Gilkerson, Glenn Browning

**Affiliations:** 1 Asia-Pacific Centre for Animal Health, Department of Veterinary Biosciences, Melbourne Veterinary School, Faculty of Veterinary and Agricultural Sciences, University of Melbourne, Parkville, VIC 3010, Australia; baileyk@unimelb.edu.au (K.B.); laura.hardefeldt@unimelb.edu.au (L.H.); jrgilk@unimelb.edu.au (J.G.); glenfb@unimelb.edu.au (G.B.); 2 National Centre for Antimicrobial Stewardship, Peter Doherty Institute, Parkville, VIC 3052, Australia; 3 ASAP Laboratory, Mulgrave, VIC 3170, Australia; bradley.galgut@asaplab.com.au; 4 Make Data Useful, Fitzroy, VIC 3065, Australia; adam.williamson@makedatauseful.com.au

**Keywords:** veterinary, antimicrobial, antibiotic, susceptibility, resistance, stewardship, guidelines, feline, canine, urine

## Abstract

International and Australian veterinary antimicrobial use guidelines recommend amoxicillin or trimethoprim-sulfonamide (TMS) for the empirical treatment of sporadic urinary tract infections (UTIs) in dogs and cats. However, in practice, these antibiotics are rarely used, and no large-scale analyses have examined the antibiograms of bacteria isolated from UTIs to validate these recommendations in Australia. We analyzed five years of urine culture and antimicrobial susceptibility data from an Australian veterinary laboratory. The analysis included 6196 urinary isolates from dogs and cats, 78% of which were from samples submitted by first-opinion veterinary clinics. *Escherichia coli*, *Enterococcus faecalis*, *Staphylococcus pseudintermedius* and *Proteus* spp. were the most prevalent organisms. More than 80% of all isolated cocci were susceptible to amoxicillin, and more than 80% of bacilli were susceptible to TMS. A total of 94% of isolates were susceptible to at least one antimicrobial drug categorized as low-importance in Australia. The prevalence of multi-drug resistance (MDR) was highest in *E. coli*, at 9.7%; 84% of these MDR isolates were susceptible to amoxicillin-clavulanate. We performed population-level antimicrobial treatment simulations and proposed a novel method for integrating antimicrobial importance ratings with antibiogram data to optimize the selection of empirical therapy. Our findings support current guideline recommendations to use amoxicillin or TMS. We also found that bacterial morphology assisted with selection; amoxicillin was a better choice for cocci and TMS for bacilli.

## 1. Introduction

Signs of lower urinary tract inflammation, such as stranguria, haematuria and pollakiuria, are a common presenting problem in dogs and cats. Although these signs are not always caused by bacterial infection, particularly in cats [1], they are a common reason for antimicrobial therapy in small animal practice [2]. Microscopy, culture and antimicrobial susceptibility testing (MCAS) is not commonly performed on initial presentation in veterinary practice. Both in Australia [3] and internationally [4], most cases of urinary tract infection (UTI) are treated empirically with antimicrobial drugs. Veterinarians are more likely to submit urine for MCAS after first-line empirical antimicrobial treatment has failed to resolve the problem or when there is a relapse [3,4]. 

The most frequently isolated organism from lower UTI in dogs and cats is *Escherichia coli*. Other Enterobacteriaceae, including *Klebsiella* spp., *Proteus* spp. and *Enterobacter* spp., are also often encountered, as are staphylococci, streptococci and enterococci [5,6,7]. 

Veterinary, agricultural and human medical organizations have developed importance ratings for antimicrobial agents to encourage greater care in the use of antimicrobials, particularly those of importance to human medicine for which there are few or no alternatives, should resistance develop [8]. In Australia, the importance ratings of the Australian Strategic and Technical Advisory Group on Antimicrobial Resistance (ASTAG) are used [9]. Antimicrobial prescribing guidelines have also proliferated. These take into account the likely pathogens, their susceptibility patterns and the best available evidence on the duration of therapy to guide empirical therapy, but only some consider antimicrobial importance ratings. International guidelines for the management of canine and feline UTIs have been available since 2011 from the International Society for Companion Animal Infectious Diseases (ISCAID), and these recommend trimethoprim-sulfonamide (TMS), amoxicillin or, where amoxicillin is unavailable, amoxicillin-clavulanate [1,10]. While the 2011 ISCAID guidelines suggested a duration of therapy of seven days, the current iteration (2019) recommends a duration of three to five days for sporadic UTIs [1]. Several national guidelines, including those in Denmark, Britain and Australia, also recommend amoxicillin or TMS [11,12,13]. However, neither of these options is commonly prescribed for UTIs in Australian cats and dogs [2] (unpublished data).

Antibiograms are tables of aggregated antimicrobial susceptibility results for isolates of a single bacterial species from one animal species over a defined period, from a particular geographical area and often a specific body site [14]. They are useful as guides for empirical treatment in the clinical setting where the organisms were found, and, when generated in a regular and consistent manner, can allow changing trends in antimicrobial resistance to be monitored [15]. The use of local and recent data to inform empirical therapy is important because of the substantial variations in microbial prevalence and antimicrobial susceptibility across geographical areas and time. To date, there have been no large-scale analyses of Australian antibiograms to evaluate the suitability of the ISCAID and Australian treatment guidelines for UTIs, and there is evidence that guidelines are not being followed, with the broad-spectrum drugs amoxicillin-clavulanate and cefovecin the most popular treatments for canine and feline UTIs in Australia [2,16]. 

This study aimed to describe the distribution, and antimicrobial susceptibility patterns, of organisms cultured from dog and cat urine samples in Australia and to formulate local empirical treatment recommendations. Recommendations can be based on two different approaches: an established method that uses only prevalence and susceptibility to calculate antimicrobial impact factors, and a novel method, used here, that also incorporates an antimicrobial importance “cost”. This novel method can help to select empirical treatment pathways that balance individual patient outcomes, which are highly visible and immediate, with the less tangible cost of antimicrobial resistance (AMR) [17]. We compared the treatment recommendations derived from these analyses with international and Australian antimicrobial prescribing guidelines for UTIs.

## 2. Results

**Figure 1:**
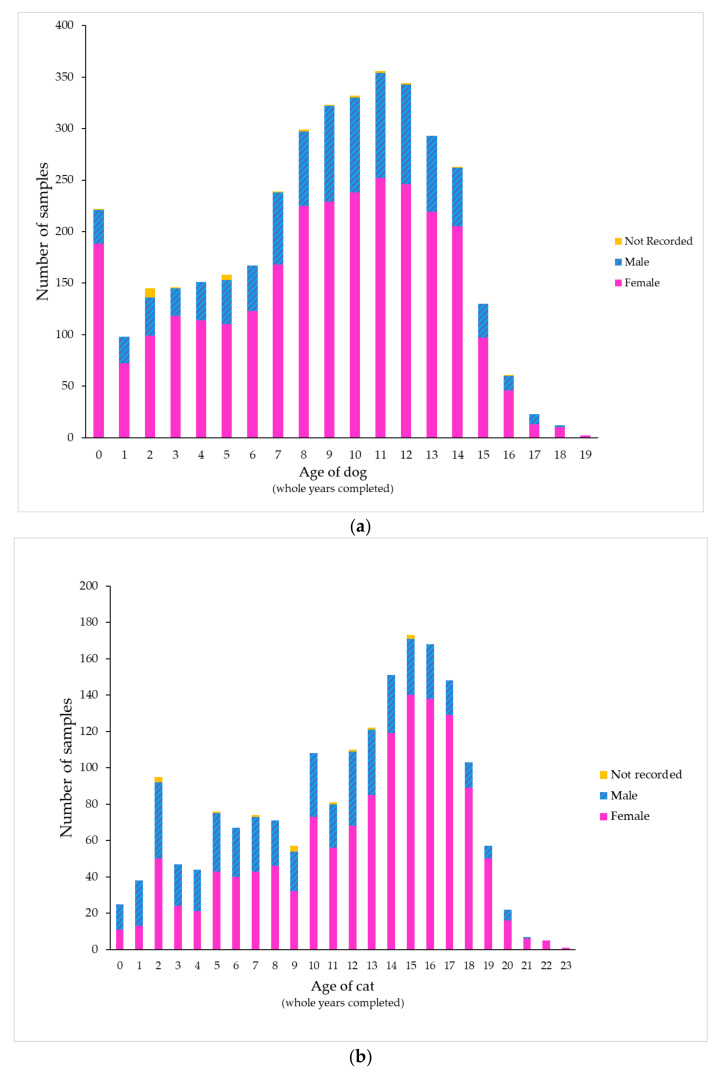
Distribution of samples by patient age and sex in (**a**) dogs and (**b**) cats.

During the study period, 263 veterinary clinics (246 first-opinion and 17 emergency/specialist practices) from a wide geographic area submitted urine samples to a commercial veterinary diagnostic laboratory. These practices included companion animal only and mixed (companion and farm animal) practices. A total of 5614 feline and canine urine samples from 4635 unique animals were present in the dataset. Twenty-two percent of the samples analyzed came from 17 emergency/specialist practices (7% of all clinics in the dataset); the remaining 78% were from first-opinion practices. Two-thirds of the samples tested came from dogs (3764 samples from 3093 dogs, 67%) and one-third came from cats (1850 samples from 1544 cats, 33%). Most samples tested from both dogs (74%) and cats (70%) were from females (*p* < 0.001). The median age was 9 years for dogs (interquartile range [IQR] 6 to 12) and 13 years for cats (IQR 7.5 to 16). In cats aged 3 years or less, males predominated (51%) compared to older cats (26%, *p* < 0.001) (Figure 1; Table S1, Supplementary Materials). While most cats were recorded as Domestic Short Hair or Domestic Long Hair, many dog breeds were represented; samples came most often from Labrador Retrievers (287 samples), followed by Pugs, Golden Retrievers and Border Collies. No clear association could be discerned between breed size and age of peak presentation (Figure S1, Supplementary Materials).

Of the 4635 animals, 627 (14%) had two or more (range 1 to 10) urine cultures in the study period, with 180 (3.9%) having three or more and 84 (1.8%) having four or more. The median interval between consecutive urine cultures on the same animal was 75 days (range 0 to 1615 days, IQR 32–211 days). Animals that had multiple urine cultures during the study period were more likely to be female than animals that had only one urine culture (dogs, OR = 1.61, 95% confidence interval [CI] 1.25–2.06, *p* < 0.001; cats, OR = 1.49, 95% CI 1.07–2.09, *p* = 0.02). There was no association between the animal’s age at the time of the first test and the probability of subsequent urine samples being submitted (*p* = 0.131). 

The method used for urine collection was recorded for less than half of all samples (44%, 2443/5614). Most labeled samples were collected by cystocentesis (1641, 29%), followed by free catch (657, 12%) and catheterization (145, 3%), with no difference between dogs and cats. Free catch samples were more likely to yield a mixed growth of organisms than samples collected by cystocentesis (OR = 2.13, 95% CI 1.52–2.98, *p* < 0.001). 

A single isolate was recorded for most of the dog (3403/3764, 90%) and cat (1730/1850, 94%) urine samples. Of the dog samples, 8% (314/3764) yielded two different isolates, 1% (42/3764) yielded three different isolates, and 0.08% (3/3764) yielded four different isolates. In cats, 6% (116/1850) yielded two different isolates, and 0.2% (3/1850) yielded three different isolates. Species other than those listed in Table 1 were found in fewer than 2% of samples, and most were found in fewer than 0.5% of samples. *Escherichia coli* (67% of cat and 55% of dog samples, *p* < 0.0001), *Enterococcus faecalis* (15% of cat and 9.3% of dog samples, *p* < 0.0001) and coagulase-negative *Staphylococcus* spp. (4.8% of cat and 2.4% of dog samples, *p* < 0.0001) were found in a higher proportion of samples from cats than samples from dogs. In contrast, *Staphylococcus pseudintermedius* (10% of dog and 7.2% of cat samples, *p* = 0.0004), *Proteus mirabilis* (11% of dog and 2.1% of cat samples, *p* = 0.0004), other *Proteus* spp. (7.7% of dog and 1.2% of cat samples, *p* < 0.0001), *Enterobacter* spp. (4.2% of dog and 1.8% of cat samples, *p* < 0.0001) and *Streptococcus canis* (2.9% of dog and 0.8% of cat samples, *p* < 0.0001) were found in a higher proportion of samples from dogs than samples from cats. 

The organisms most commonly cultured as a pure growth were coagulase-negative staphylococci (92%) and *E. coli* (90%) (Table 1). Those most frequently found as a component of a mixed growth were *E. faecalis* (61%) and *S. canis* (62%). Where *E. faecalis* was cultured in mixed growth, it was most frequently found with *E. coli* (149/254, 59%), followed by *S. pseudintermedius* (41/254, 16%) and *P. mirabilis* (31/254, 12%).

**Figure 2:**
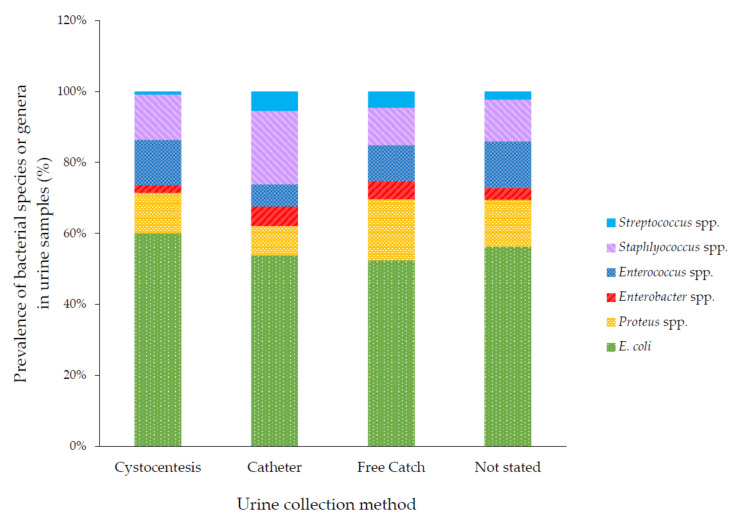
Bacterial species cultured, by collection method. Some totals are >100% because some samples yielded multiple bacterial species.

The types of organisms grown were associated with the method of sample collection (*p* < 0.001). Catheterized samples were the most likely sample type to yield *Enterobacter* spp., *Staphylococcus* spp. and *Streptococcus* spp. Free catch samples were the most likely to yield *Proteus* spp. (Figure 2).

Most bacterial isolates in this study (5843/6196, 94%) were susceptible to one or more antimicrobials regarded as having low importance by the ASTAG (amoxicillin, TMS, doxycycline, erythromycin or tetracycline). Of those resistant to these drugs, more than two-thirds (250/353, 71%) were susceptible to an antimicrobial agent regarded as having medium importance by the ASTAG, amoxicillin-clavulanate. One percent (60/5614) of samples yielded isolates resistant to all low- and medium-importance agents tested. Of these, two-thirds (40/5614, 0.7%) were susceptible to enrofloxacin, but none was susceptible to cefovecin. More than 80% of the isolates of each species or genus were susceptible to each of the drugs tested (Table 2 and Table 3). However, in both dogs and cats, *E. faecalis* showed low susceptibility to enrofloxacin (0.9% and 1.4% susceptible respectively), tetracycline (54% and 53% susceptible respectively) and erythromycin (76% and 77% susceptible). There were too few *S. canis* isolates from cats (14 isolates) for further analysis, however, in dogs (111 isolates) extensive resistance to enrofloxacin (95% resistant) and high-level resistance to gentamicin (97% resistant) were identified. 

### 2.1. Antimicrobial Impact Factor

The impact factor was calculated using the formula for rational antimicrobial therapy (sum of % prevalence X% susceptible for organism 1 to *n*) for all species/genera found in at least 2% of cases [18]. This factor is an estimate of the percentage of UTI cases that are expected to respond to that antimicrobial when all cases are treated empirically. 

**Figure 3:**
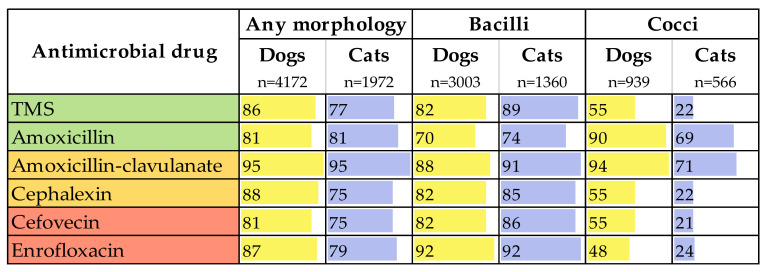
Impact factors of the six routinely tested antimicrobials, by isolate morphology and host species. The colors in the left column indicate the Australian Strategic and Technical Advisory Group on Antimicrobial Resistance (ASTAG) importance ratings of the antimicrobials, and the yellow and blue bars indicate the magnitude of the antimicrobial impact factor in each animal species.

The antimicrobials with the highest impact factors for dogs were amoxicillin-clavulanate (95), enrofloxacin (87), TMS (86) and amoxicillin (81). In cats, amoxicillin-clavulanate (95) was also ranked highest, followed by amoxicillin (81) and enrofloxacin (79), closely followed by TMS (77) (Figure 3).

When the organisms were sorted by morphology (bacilli versus cocci), enrofloxacin had the highest impact factor for bacilli (92 in dogs and cats), however, amoxicillin-clavulanate (88 in dogs and 91 in cats) and TMS (82 in dogs and 89 in cats) also had high impact factors. For cocci, antimicrobial impact factors were lower across the board for cats, compared with dogs. Amoxicillin-clavulanate had the highest impact factor for both species (94 in dogs and 71 in cats), but amoxicillin (90 in dogs and 69 in cats) also had high impact factors.

### 2.2. Whole-Population Antimicrobial Simulation and Antimicrobial Cost per Cure

**Figure 4:**
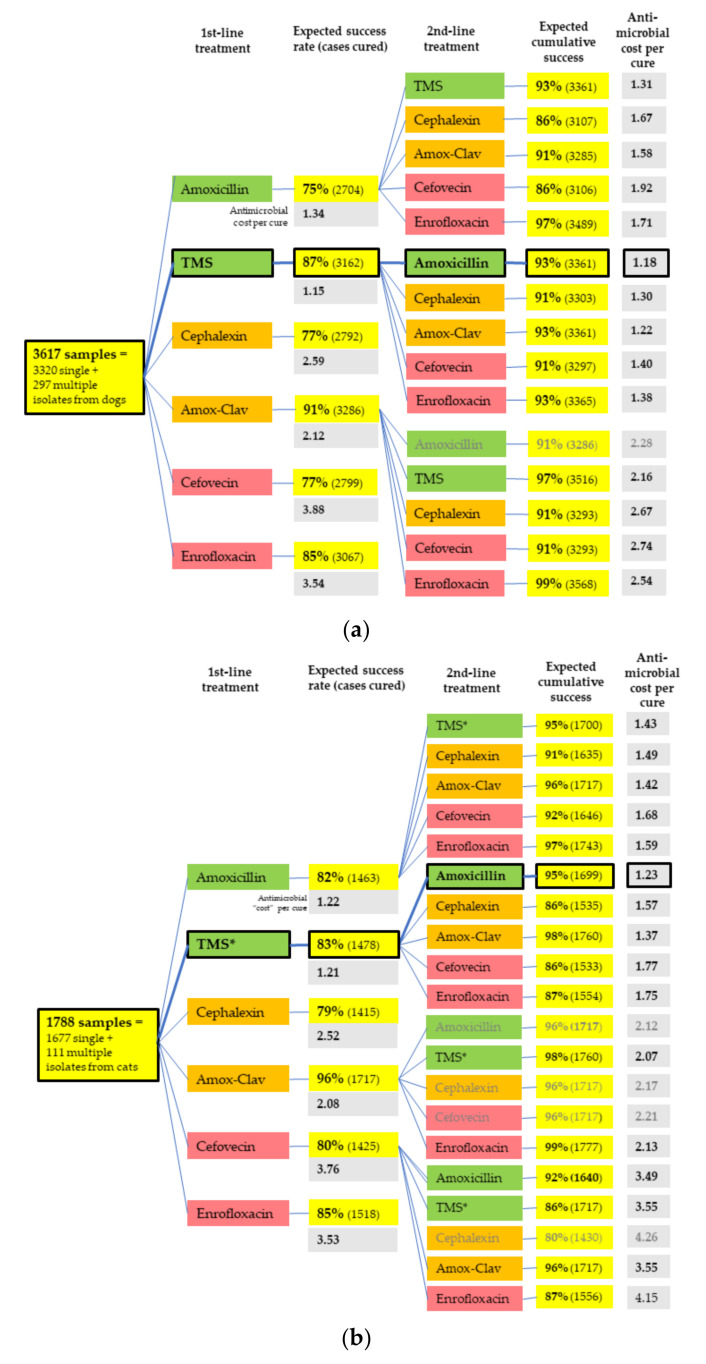
Whole population treatment simulation, including antimicrobial cost per cure in (**a**) dogs and (**b**) cats. Amox-Clav = amoxicillin-clavulanate. Bold outlines indicate the best pathways by antimicrobial cost. Colors indicate the ASTAG importance ratings of antimicrobials [9], where green are the least important (preferred choices), and red are the most important (least preferred choices), due to the risk that selection of resistance poses to human and animal health. Only selected second-line pathways are displayed—those with the lowest antimicrobial cost per cure and those following first-line choices that are most commonly used for UTIs in that animal species in Australia—amoxicillin-clavulanate in dogs and cefovecin and amoxicillin-clavulanate in cats [2] (unpublished data).

By accounting for the importance rating of each antimicrobial and the number of animals treated in each pathway, the treatment pathway with the lowest antimicrobial “cost” per cure for both dogs and cats was determined. This pathway was found to comprise initial treatment with TMS, followed by amoxicillin should this initial therapy fail (Figure 4). 

The proportions of each species/genus of bacteria recovered from urine samples with no acquired resistance or with MDR are shown in Table 4. MDR had the highest prevalence in *E. coli* (320/3294, 9.7%), most commonly involved beta-lactam resistance, but 268/320 (84%) of these MDR isolates were susceptible to amoxicillin-clavulanate. One *E. coli* isolate was resistant to ten antimicrobials tested (Table S2, Supplementary Materials). Resistance patterns for *E. faecalis*, *S. pseudintermedius* and *P. mirabilis* are also included in Supplementary Materials (Tables S3–S5, Supplementary Materials). Oxacillin testing was not performed on *S. pseudintermedius* isolates, therefore the rate of methicillin resistance is unknown. As *Enterobacter* spp. are intrinsically resistant to most of the antimicrobials routinely tested at this laboratory, we could only assess susceptibility to TMS and enrofloxacin. Eighty-three percent (157/190) of the *Enterobacter* spp. isolates were susceptible to both these drugs. Thirty isolates (16%) were resistant to TMS, seven (3.7%) were resistant to enrofloxacin, and four (2.1%) were resistant to both. No association was found between the animal’s age and the antimicrobial susceptibilities of the most common isolates.

### 2.3. Changes in Resistance

**Figure 5:**
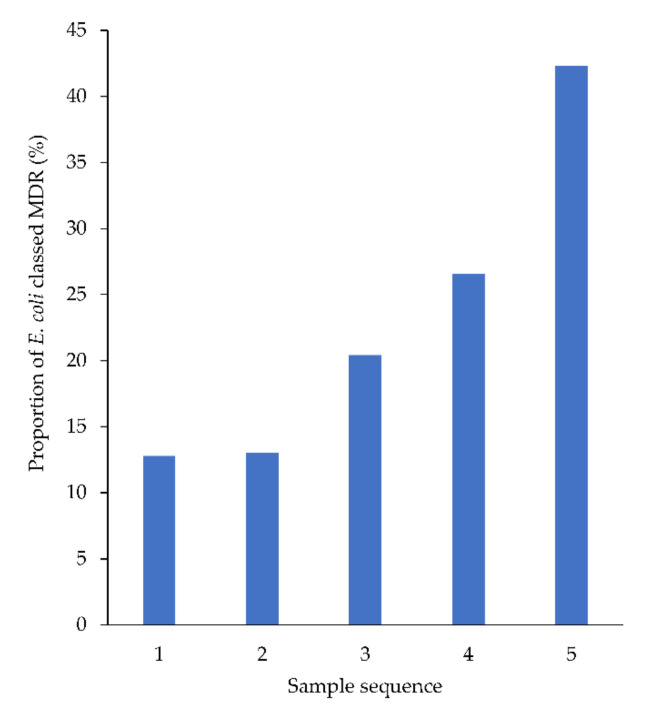
Proportion of *E. coli* (from animals from which ≥4 samples were cultured) classed as MDR, by sample sequence number.

No changes in resistance patterns were detected across the population over time (Figure S2a–g, Supplementary Materials). However, in animals from which four or more urine samples were cultured during the study period, the proportion of *E. coli* classified as MDR increased by 7.3% with each successive sample, from sample 1 to 5 (95% CI: 4.3% to 10%) (Figure 5).

## 3. Discussion

In general, little acquired antimicrobial resistance was observed in canine and feline urinary tract pathogens in this large Australian sample. The vast majority of the bacterial isolates were susceptible to the guideline-recommended antimicrobials, amoxicillin or TMS, and most bacteria isolated were susceptible to at least one of the antimicrobials tested that is classified as having low importance by the ASTAG (94% susceptible to one of amoxicillin, TMS, doxycycline, tetracycline or erythromycin). Two-thirds of the remainder (4%) were susceptible to amoxicillin-clavulanate.

These results should be considered to underestimate the effectiveness of these antimicrobials in typical canine and feline UTI presentations for three reasons. Firstly, since veterinarians are more likely to submit urine samples for MCAS after first-line empirical antimicrobial treatment has failed to resolve the infection, or when there is a relapse [3], the isolates represented in these antibiograms are likely to have had previous exposure to antimicrobial therapy, increasing their chance of resistance [19], and more complicated UTI cases are probably over-represented. Secondly, the cutoffs used by the laboratory to determine whether an isolate is susceptible or resistant are based on drug concentrations achieved in plasma and tissues. However, some antimicrobials—including penicillins, cephalosporins and quinolones [20]—reach considerably higher concentrations in the urine. This means that our results could be considered to underestimate the in vivo effectiveness of some of the antimicrobials in the lower urinary tract. Thirdly, in vivo cure rates for all antimicrobials are likely to be higher than in vitro susceptibility would suggest, as laboratory results do not account for the activity of the animal’s immune response, which augments the effect of antimicrobial therapy [21]. Thus, the actual UTI cure rate (as well as the antimicrobial impact factors) would probably be higher than we have assumed.

Almost three-quarters of samples from both dogs and cats were from females, consistent with the higher incidence of UTIs in females of many species [22]. However, in cats under three years of age, male cats outnumbered female cats, perhaps because idiopathic feline lower urinary tract disease (FLUTD)—which is sometimes accompanied by bacterial UTI [23]—is more common in young male cats [24]. This pattern of younger males and older females was also seen in a German study of cat UTIs. [25]

*E. coli* was the most prevalent species isolated from dog and cat urine samples in this study (59% of samples), similar to international studies [6,26,27], but substantially higher than in another study from Australia [7] and one from New Zealand [28]. *E. faecalis* (11.5%), *S. pseudintermedius* (9.2%), *P. mirabilis* (7.9%), other *Proteus* spp. (5.5%), *Enterobacter* spp. (3.6%), coagulase-negative staphylococci (3.2%) and *S. canis* (2.2%) were also detected in significant proportions of cases, as reported elsewhere [5,27,28]. Notably, the proportion of MDR *E. coli* in this study (9.7%) was somewhat lower than in a previous Australian study, which found 16% of *E. coli* isolated from canine urine samples and 11% of *E. coli* isolated from feline urine samples to be MDR. However, this earlier study included fewer urinary tract isolates (*n* = 672), and the samples were from university and government laboratories [29], and thus a greater proportion may have come from more complex and refractory cases. Globally, there is substantial variation in resistance patterns among *E. coli* isolates from canine and feline urinary tract disease. In the USA, approximately 46% of *E. coli* isolates were resistant to amoxicillin in two studies [6,30], compared with 23% of the isolates in our study, and the prevalence of resistance to amoxicillin-clavulanate as high as 35% [30], compared with 2% in our study. Another U.S. study found that only 35% of canine urinary *E. coli* isolates were susceptible in vitro to the first-generation cephalosporin, cefazolin [31], while 90% of the isolates in our study were susceptible to cephalexin. While one of these U.S. studies found no oral antimicrobial agent that was effective in vitro against more than 90% of their isolates of *E. coli* from UTIs [6], our study found six oral antimicrobials for dogs and five for cats that had more than 90% efficacy against urinary tract *E. coli*. In Europe, resistance to amoxicillin-clavulanate ranges from 2.9% (Sweden) to 32% (Spain) and MDR rates from 0.24% (Sweden) to 30% (Spain) [27]. High rates of resistance have also been detected in South African (98% MDR, 11% extensively drug-resistant, 2% pan-drug-resistant) [28] and Brazilian (66% MDR) [32] urinary tract *E. coli* from dogs. These differences highlight the need for the determination of local antibiograms to assist in the development and validation of local antimicrobial prescribing guidelines.

The canine and feline UTI isolates examined in our study were more susceptible than recent Australian UTI isolates from human patients to both amoxicillin (77% in dogs and cats vs. 54% in humans) and amoxicillin-clavulanate (97% in dogs and cats vs. 87% in humans) [33]. However, a potentially concerning finding in our study was the high level of fluoroquinolone resistance in *E. faecalis*. Although enterococci have relatively low inherent susceptibility to fluoroquinolones [34], resistance to enrofloxacin among *E. faecalis* isolates was nearly universal throughout the study period (99%). This level of fluoroquinolone resistance in enterococci has not been reported previously in Australia and is much higher than that detected in the USA (17/35, 49%) [35] and New Zealand (79/280, 28%) [28]. However, there is some uncertainty about the pathogenicity of enterococci in the canine and feline urinary tract. Although acknowledged as an important urinary pathogen in humans [33] and known to invade urinary epithelial cells [36], enterococci have commonly been considered an incidental finding in dog and cat urine. This is partly because enterococci are frequently found in mixed cultures [27,37] and are often found in the urine of animals without clinical signs of UTI [35]. One author has stated that the “low pathogenicity of *Enterococcus* may not justify the risks and expense of antibiotic treatment” [38] (p.1094). When enterococci are present in mixed bacterial cultures (as for almost 40% of all samples that yielded enterococci in this study), treatment aimed at the other bacterial species may result in clearance of enterococci without targeting therapy at these species [10,38].

*S. canis* isolates in this study were also almost universally resistant to enrofloxacin (114/119, 96%). In contrast, in a New Zealand study, only 2% of beta-hemolytic streptococci were found resistant to enrofloxacin [28]. Genetic studies of *E. faecalis* and *S. canis* from local animal samples would be helpful to determine the mechanism and origin of enrofloxacin resistance. Enrofloxacin, marbofloxacin and pradofloxacin are registered for use in dogs and cats in Australia [16]. Additionally, fluoroquinolones such as ciprofloxacin have been widely used in human medicine in Australia in the past and are increasingly prescribed to treat human UTIs. A 2011 trial in dogs found high-dose treatment with enrofloxacin for three days was effective for dog UTIs [39]. However, because of their high importance in both veterinary and human medicine, fluoroquinolones should be reserved for cases where MCAS demonstrates that no antimicrobials of lower importance are effective.

To avoid bacterial contamination from the urethra, perineum and other surfaces, cystocentesis samples are always preferable for MCAS, but these may be contraindicated in some circumstances and are not always readily obtained. This is reflected by the significant proportion of samples that were labeled as free catch (voided) or collected via a catheter in our study. Unsurprisingly, the urine collection method affected both the proportions of each of the bacterial genera cultured, as well as the likelihood of a mixed culture, as found in other studies [25]. This underlines the need for considered clinical interpretation of urine MCAS results, especially when a voided sample has been used.

A significant proportion of animals (627, 14%) had more than one urine MCAS performed by this laboratory in the study period. Although treatment data for these animals was not available, this subset can be reasonably assumed to represent animals that were treated with antimicrobials after their last MCAS and then returned to the veterinarian because of treatment failure, relapse or reinfection. The increased prevalence of MDR *E. coli* with each subsequent sample (from 8% in first samples to 29% in fourth samples) suggested that the intervening antimicrobial therapy had selected for MDR determinants, consistent with findings from other studies [29,35].

Accepting the previously mentioned limitations of our susceptibility data, the impact factors calculated for each antimicrobial [18] suggest that amoxicillin-clavulanate is the most rational choice for the empirical treatment of UTIs. However, this potentiated penicillin is a medium-importance antimicrobial, therefore agents with lower importance should be chosen where clinically appropriate to reduce the risk of selection for antimicrobial resistance. Amoxicillin and TMS are both low-importance antimicrobials with high efficacy against the urinary isolates examined in this study, and we demonstrated that bacterial morphology clearly determines differing superior choices: TMS for bacilli and amoxicillin for cocci. Most veterinary clinics in Australia are equipped for this point-of-care determination. If bacilli are identified, the impact factor of TMS approaches that of amoxicillin-clavulanate (82 vs. 88 in dogs and 89 vs. 91 in cats); a treatment duration of three to five days is recommended [1,12], and a trial in female dogs showed that three days of TMS was not inferior to 10 days of cephalexin [40]. Susceptibility results should always be interpreted by veterinarians in combination with their clinical judgment and consideration of relevant risk factors for individual patients. A number of adverse reactions to TMS have been reported in dogs and are outlined in the relevant guidelines. These include dose-dependent reactions, such as keratoconjunctivitis sicca and thyroid dysfunction, and delayed hypersensitivity reactions, including hepatopathy, blood dyscrasias and polyarthritis, which typically occur with courses of more than five days of therapy [41,42,43]. Challenges posed by hypersalivation in cats with oral TMS [44] can be overcome by using intact (not split) coated tablets, which are available in Australia and elsewhere. If cocci are identified, the expected efficacy of amoxicillin is very close to that of amoxicillin-clavulanate (antimicrobial impact factors of 90 vs. 94 in dogs, 69 vs. 71 in cats). In other words, 27 dogs or 40 cats would need to be treated with amoxicillin-clavulanate to achieve one additional cure, compared with amoxicillin.

Our novel population-level antimicrobial simulation, which accounts for the difference in antimicrobial importance ratings as well as the local prevalence of the different pathogens and their in vitro susceptibility, suggests that, where morphology and Gram stain are unknown, the most rational and appropriate first-line empirical choice is TMS, and, where this is not effective or clinically appropriate because of the risks of adverse effects, the most rational and appropriate second-line choice is amoxicillin. These findings support both the ISCAID [1] and the Australian Veterinary Prescribing Guidelines [12]. The use of evidence-based scores for AMR risk for each antimicrobial treatment, rather than the empirical scores of 1 to 3 applied in our study, would improve this simulation. It would also be valuable to repeat the antimicrobial simulation using Clinical and Laboratory Standards Institute (CLSI) susceptibility results and urine-specific breakpoints.

One limitation to our study is the laboratory’s use of the calibrated dichotomous sensitivity (CDS) method and its standard practice of only recording a binary result—susceptible or resistant—rather than a radius of inhibition. This means we cannot estimate minimum inhibitory concentrations, or make adjustments for the high urine concentration of some antimicrobial drugs. The lack of oxacillin/cefoxitin testing of staphylococci also means that we have been unable to assess the prevalence of methicillin resistance, which is an important indicator for both AMR surveillance and clinical management. Another limitation was the absence of data on bacterial concentrations in urine samples, as well as corresponding clinical data and urinalysis results for these cases, prohibiting triangulation of susceptibility data with clinical signs and prior and subsequent antimicrobial therapy.

## 4. Materials and Methods

### 4.1. Source and Handling of Data

De-identified data were obtained from a veterinary diagnostic laboratory in Australia. Microscopy, culture and antimicrobial susceptibility test results from all cat and dog urine samples that were submitted to the laboratory over a 5-year period (1 January 2015 to 31 December 2019) were included in the study. A unique numerical code was assigned by the laboratory to each patient at the de-identification stage to allow recognition of multiple submissions from the same patient. Culture-negative results were excluded from the analysis. For organisms with intrinsic resistance to an antimicrobial (including in vivo resistance despite in vitro susceptibility), any “susceptible” laboratory results were altered to resistant. Urinalysis and other clinical data were not available.

### 4.2. Sample Processing

Antimicrobial susceptibility testing was performed by the laboratory using the standardized calibrated dichotomous sensitivity (CDS) method [45] by inoculation of urine samples directly onto agar plates then adding the antimicrobial discs: ampicillin 10 µg (reported as amoxicillin), amoxicillin/clavulanate 20/10 µg, cephalexin 30 µg, cefovecin 30 µg, enrofloxacin 5 µg and sulfamethoxazole/trimethoprim 1.25/23.75 µg. Some isolates were tested against additional antimicrobials—including marbofloxacin, clindamycin and tetracycline—either in response to special requests from the treating veterinarian or because of antimicrobial resistance seen in the initial tests. For enterococcal isolates, additional antimicrobial discs were applied: erythromycin 15 µg, doxycycline 30 µg and gentamicin 120 µg (to detect high-level resistance). The radius of the zone of inhibition was not recorded. Results were reported as either susceptible or resistant to each antimicrobial drug.

### 4.3. Antibiogram, Antimicrobial Impact Factors and Antimicrobial Cost per Cure

The eight most prevalent bacterial species/groups were included in the antibiograms. As not all isolates were tested against the same antimicrobials, results were calculated as the susceptible proportion of the isolates tested against that antimicrobial. Groups of fewer than 30 isolates were excluded from calculations of antimicrobial impact factors and from resistance charts. Multi-drug resistance (MDR) was defined as non-intrinsic resistance to three or more of the antimicrobial categories tested. Antimicrobial categories were based on definitions published previously [18], with the addition of aminopenicillins, first-generation cephalosporins, third-generation cephalosporins and beta-lactam inhibitor combinations for staphylococci.

The formula for the selection of rational antimicrobial therapy [18] was applied to bacterial species that were present in at least 2% of urine samples from that host species to derive an “impact factor” for each antimicrobial agent. This is an estimate of the proportion of overall UTI cases expected to respond to empirical therapy with that antimicrobial. The impact factor calculation was repeated, differentiating isolates as bacilli and cocci; most veterinary clinics in Australia are equipped for the microscopy of urinary sediment, making morphology of bacteria a feasible approach to the selection of empirical therapy.

To further explore rational treatment pathways, we simulated the theoretical population-level effect of using two antimicrobials in sequence on the dog and cat urine samples that had been tested against all six antimicrobials in the laboratory’s standard panel, based on the in vitro susceptibility of the isolate/s in each sample. Treatment was considered successful once all the isolates in a single sample had been eliminated, or where the only remaining isolate from a sample containing multiple species was an *Enterococcus* spp. Each treatment course of the six antimicrobials was allocated an antimicrobial cost, according to the antimicrobial’s importance rating from the Australian Strategic and Technical Advisory Group on Antimicrobial Resistance [9], with the highest importance antimicrobials given a cost score of three points, those with medium importance a score of two points and those with a low importance a score of one point. The antimicrobial cost per cure for each treatment pathway was then calculated as follows:

$$\frac{Number\;of\;cases\;treated\;\times antimicrobial\;cost\;per\;treatment\;course}{Number\;of\;cases\;cured}$$

Data were cleaned using Python and Microsoft Excel, and descriptive statistics were generated in Microsoft Excel. Regressions and tests of proportions were performed using R Base Package [46], dplyr [47], tidyverse [48] and epiDisplay [49]. The desexing status was inconsistently recorded, therefore spayed and entire females were combined, as were castrated and entire males.

## 5. Conclusions

Our study provided much-needed antimicrobial susceptibility data and practically applicable insights to assist small animal practitioners in the treatment of UTIs. This five-year sample, largely from first-opinion veterinary practices, showed relatively little acquired antimicrobial resistance overall, and very little multiple drug resistance, in urinary bacterial isolates from Australian dogs and cats. However, there were high levels of resistance to fluoroquinolones in *E. faecalis* and *S. canis*. For complicated cases, where animals had multiple urine cultures performed, there was a significant increase in the prevalence of MDR *E. coli* with each successive culture. These findings underscore the impact of veterinary antimicrobial prescribing on resistance patterns and the need for veterinarians to dispense antimicrobials with care.

The antibiograms and rational antimicrobial therapy simulation support current international and Australian guideline recommendations to use three to five days of TMS or amoxicillin as the first-line, empirical therapy for dog and cat UTIs. These agents remain effective against more than 80% of urinary isolates and pose a lower risk of selection for significant AMR than most of the other agents tested. The use of fluoroquinolones (which have a high importance rating) was microbiologically justified in only 0.7% of cases, while the use of the high-importance, third-generation cephalosporin, cefovecin, was not microbiologically justified for any cases.

## Figures and Tables

**Table 1:** Organisms cultured for each collection method and proportion cultured as a pure growth.

Organism Species or Group	% of All Samples	Urine Collection Method, *n* (% as Pure Growth)				
		Cystocentesis	Catheter	Free Catch	Not Stated	Total
*Escherichia coli*	59%	1004 (94)	78 (89)	357 (86)	1855 (90)	3294 (90)
*Enterococcus faecalis*	11%	192 (72)	8 (63)	63 (54)	373 (55)	636 (61)
*Staphylococcus pseudintermedius*	9.1%	155 (92)	22 (96)	55 (87)	281 (75)	513 (87)
*Proteus mirabilis*	7.8%	110 (86)	8 (100)	72 (81)	250 (85)	440 (88)
*Proteus* spp.	5.5%	80 (89)	4 (50)	43 (88)	183 (78)	310 (76)
*Enterobacter* spp.	3.4%	36 (91)	8 (100)	35 (88)	111 (80)	190 (88)
Coagulase-negative *Staphylococcus* spp.	3.2%	55 (91)	8 (100)	16 (88)	98 (88)	177 (92)
*Streptococcus canis*	2.2%	13 (69)	8 (75)	31 (55)	73 (50)	125 (62)
All other organisms	8.2%	82	13	55	309	459

**Table 2:** Antimicrobial susceptibility and importance ratings for the most prevalent urinary isolates from dogs. Amox: amoxicillin; TMS: trimethoprim-sulfonamide; Tetra: tetracycline; Doxy: doxycycline; Erythro: erythromycin; Gentam: Gentamicin; Amox-Clav: amoxicillin-clavulanate; Ceph: cephalexin; Enroflox: enrofloxacin.

			Low-Importance Antimicrobials					Medium-Importance Antimicrobials			High-Importance Antimicrobials	
Organism	*n*	% of Dog Isolates	Amox	TMS	Tetra	Doxy	Erythro	Gentam	Amox-Clav	Ceph	Cefovecin	Enroflox
*Escherichia coli*	2058	55%	75%	93%	82%	98%	IR	93%	97%	90%	90%	95%
*Proteus mirabilis*	401	11%	91%	94%	IR	IR	IR	100%	99%	95%	97%	99%
*Staphylococcus pseudintermedius*	380	10%	91%	93%	76%	85%	75%	89%	98%	95%	95%	98%
*Enterococcus faecalis*	351	9.3%	96%	IR	54%	91%	76%	95% *	95%	IR	IR	0.9%
*Proteus* spp.	288	7.7%	87%	92%	1.6% **	0% **	IR	100%	98%	96%	94%	98%
*Enterobacter* spp.	157	4.2%	IR	86%	86%	100%	IR	100%	IR	IR	IR	96%
*Streptococcus canis*	111	2.9%	96%	96%	84%	97%	89%	IR	97%	90%	89%	4.8%
Coagulase-negative *Staphylococcus* spp.	89	2.4%	87%	93%	86%	86%	93%	95%	97%	94%	92%	98%

* Enterococci were tested against a higher-potency gentamicin disc (120 µg) to detect high-level resistance. ** Some *Proteus* spp. are intrinsically resistant, and others are not, but it was not possible to separate species with the data available. IR = intrinsic resistance. Gray = fewer than 30 isolates of this species/genus were tested against this antimicrobial, and/or this species/genus is intrinsically resistant to this antimicrobial; Green = more than 80% of isolates susceptible; Yellow = 60%−80% isolates susceptible; Pink = less than 60% of isolates susceptible.

**Table 3:** Antimicrobial susceptibility and importance ratings for the most prevalent urinary isolates from cats. Amox: amoxicillin; TMS: trimethoprim-sulfonamide; Tetra: tetracycline; Doxy: doxycycline; Erythro: erythromycin; Gentam: Gentamicin; Amox-Clav: amoxicillin-clavulanate; Ceph: cephalexin; Enroflox: enrofloxacin.

			Low-Importance Antimicrobials					Medium-Importance Antimicrobials			High-Importance Antimicrobials	
Organism	*n*	% of Cat Isolates	Amox	TMS	Tetra	Doxy	Erythro	Gentam	Amox-Clav	Ceph	Cefovecin	Enroflox
*Escherichia coli*	1236	67%	80%	94%	82%	82%	IR	100%	99%	92%	93%	97%
*Enterococcus faecalis*	285	15%	98%	IR	53%	87%	77%	95% *	99%	IR	IR	1.4%
*Staphylococcus pseudintermedius*	133	7.2%	91%	97%	89%	100%	100%	100%	100%	93%	94%	100%
Coagulase-negative *Staphylococcus* spp.	88	4.8%	98%	100%	100%	100%	100%	100%	99%	98%	98%	100%
*Proteus mirabilis*	39	2.1%	87%	92%	IR	IR	IR	0.0%	100%	95%	95%	97%
*Enterobacter* spp.	33	1.8%	IR	76%	100%	100%	IR	100%	IR	IR	IR	97%
*Proteus* spp.	22	1.2%	82%	73%	0% **	0% **	IR	100%	96%	87%	82%	100%
*Streptococcus canis*	14	0.8%	100%	86%	86%	100%	100%	IR	100%	100%	100%	0%

* Enterococci were tested against a higher-potency gentamicin disc (120 µg) to detect high-level resistance. ** Some *Proteus* spp. Are intrinsically resistant, and others are not, but it was not possible to separate species with the data available. IR = intrinsic resistance. Gray = fewer than 30 isolates of this species/genus were tested against this antimicrobial, and/or this species/genus is intrinsically resistant to this antimicrobial; Green = more than 80% of isolates susceptible; Yellow = 60%−80% isolates susceptible; Pink = less than 60% of isolates susceptible.

**Table 4:** Summary of antimicrobial resistance detected in the most prevalent bacterial species/genera isolated from dog and cat urine. MDR: multi-drug resistance.

Organism	Total Number of Isolates	Intrinsic Resistances	Isolates with no Acquired Resistance Detected *n* (%)	MDR = Resistance to Three or More of:	MDR *n* (%)	Most Common MDR Combination (*n*)	Prevalence of Most Common MDR Combination ^##^ *n* (%)
*Escherichia coli*	3294	-	2450 (74)	AMX, AMC, LEX, CVN, TET, GEN, FQN, SXT	320 (9.7)	AMX + LEX + CVN	276 (8.4)
*Enterococcus faecalis*	636	LEX, CVN, SXT	22 (3.5)	AMX, TET, GEN*, ERY, FQN	19 (3.0)	TET + ERY + FQN	8 (1.3)
*Staphylococcus pseudintermedius*	513	-	433 (84)	AMX, AMC, LEX, CVN, TET, GEN, FQN, SXT	34 (6.6)	AMX + LEX + CVN	28 (5.5)
*Proteus mirabilis*	440	TET	378 (86)	AMX, AMC, LEX, CVN, FQN, SXT	16 (3.6)	AMX + LEX + CVN	13 (2.9)
*Proteus* spp.	310	^#^	249 (80)	AMX, AMC, LEX, CVN, FQN, SXT	16 (5.2)	AMX + LEX + CVN	8 (2.6)
*Enterobacter* spp.	190	AMX, AMC, LEX, CVN	157 (83)	Not assessable **			
Coagulase-negative *Staphylococcus* spp.	177	-	155 (88)	AMX, AMC, LEX, CVN, TET, GEN, FQN, SXT	8 (4.5)	AMX + LEX + CVN	6 (3.4)
*Strep. canis*	125	GEN	8 (6.4)	AMX, AMC, LEX, CVN, TET, GEN, FQN, SXT	2 (1.6)	AMX + FQN + SXT	1 (0.8)

AMX = amoxicillin (tested using ampicillin disc); AMC = amoxicillin-clavulanate; LEX = cephalexin; CVN = cefovecin; FQN = any fluoroquinolone (enrofloxacin most commonly tested); SXT = trimethoprim-sulfamethoxazole (TMS); ERY = erythromycin; GEN = gentamicin; TET = tetracyclines (doxycycline, tetracycline). *Note:* Not all isolates were tested against all antimicrobials. * Enterococci tested against a higher-potency gentamicin disc (120 µg) to detect high-level resistance. ** MDR cannot be assessed as only two antimicrobials (TMS, ENR) were routinely tested, apart from those to which *Enterobacter* spp. are intrinsically resistant. ^#^ Some *Proteus* spp. (other than *P. mirabilis*) have intrinsic resistance to TET, but the laboratory did not identify to the species level. ^##^ Includes isolates that have this combination plus additional resistances.

## Supplementary Materials

The following are available online at https://www.mdpi.com/2079-6382/9/12/924/s1, Figure S1: Frequency of samples by dog breed and age, Figure S2: Changes in susceptibility over time for the most common species/genera, Table S1: Urine sample source by animal species, age and sex, Table S2: Multiple resistance patterns in *E. coli*, Table S3: Multiple resistance patterns in *Enterococcus faecalis*, Table S4: Multiple resistance patterns in *Staphylococcus pseudintermedius*, Table S5: Multiple resistance patterns in *Proteus mirabilis.*
